# An understanding of third-party friendships in a tolerant macaque

**DOI:** 10.1038/s41598-020-66407-w

**Published:** 2020-06-17

**Authors:** Jamie Whitehouse, Hélène Meunier

**Affiliations:** 1 Centre de Primatologie, de l’Université de Strasbourg, Niederhausbergen, France; 2 Laboratoire de Neurosciences Cognitives et Adaptatives, UMR 7364,, Université de Strasbourg, Strasbourg, France

**Keywords:** Evolution, Psychology

## Abstract

Complex societies are shaped by social relationships between multiple individuals. The pressure to track these relationships has driven the evolution of social cognition in primates. Importantly, it can be adaptive to track not only personal relationships, but also those established between third-parties. Primates have knowledge about others’ dominance hierarchies and kinship, but we do not know to what extent they also understand friendships. In a playback experiment, Tonkean macaques were presented with simulated conflicts involving third-party female dyads who were established friends or non-friends. Hearing a conflict between friends elicited a stronger behavioural response in listeners (i.e. an increase in looking time) compared to hearing a conflict between non-friends. Conflicts between friends are likely to represent a greater disruption of the social group and structure of the network, and therefore this difference in response may represent an adaptive strategy employed by the macaques to selectively monitor important social interactions in the group. These findings provide evidence that Tonkean macaques (and potentially other primates) can classify the relationships of others based on their degree of friendship and additionally, confirms the important role friendships have within the societies of social primates.

## Introduction

Many primates, including humans, live in complex societies shaped by the social relationships that they form with others. These societies are built around networks of social relationships, where pairs of individuals develop unique social connections due to relatedness^1^, dominance^2^ and friendship (i.e. strong positive asssociation between individuals^3^). The adaptive value of social relationships in maintaining stable and cohesive social groups has driven the evolution of social knowledge in primates^4^, and has allowed for a cognitive capacity that enables primates (and many non-primates) to retain information about the relationships they have previously established with others^5^. This ability has been evidenced in numerous observational and playback experiments^6^, where monkeys have been shown to look preferentially and respond adaptively to the social stimuli of their kin^7^, friends^8^ or more dominant individuals^9^. The extent of this social knowledge however, seems not only limited to an individuals’ direct connections, but extends to third-party social relationships in their group that they themselves are not directly involved in^10^.

Monkeys have been shown to classify the relationships of others based on both relatedness and dominance. For example, vervet monkeys (*Chlorocebus pygerythrus*) will attend to the mothers of distressed infants^7^, and long-tailed macaques (*Macaca fascicularis*) were able to correctly match mother-infant pairs when classifying images^11^. Japanese macaques (*Macaca fuscata*) selectively recruit individuals who are higher-ranking than their opponents as allies during conflicts^12^, and baboons (*Papio hamadryas ursinus)* have been demonstrated to classify third-party social relationships based on kin and rank in combination, and are sensitive to third-party rank-reversals that may impact whole family groups^13^. Beyond the classification of rank and kin, there is evidence to suggest that baboons (*Papio papio*) living in multi-level societies understand the unit affiliation of females^14^. Additionally, non-human primates attend and monitor transient relationships between ovulating females and males during consortships^15,16^ and eavesdrop on mating pairs^17^. We would therefore expect that more stable social relationships, such as those developed through friendships, would also be monitored and understood by primates (as longer, more stable relationships should be easier to learn over time).

Maintaining close friendships is an highly adaptive strategy, with both long-term benefits such as a greater likelihood for offspring to survive, increased longevity^18,19^ and a better ability to cope with stress^20^, and immediate benefits such as cooperation during anti-predator behaviour^8^. Individuals seem to not only gain fitness from their own, direct friendships, but from their indirect ‘friend-of-friends’ connections, as these represent potential social pathways to the rest of the social network^21,22^. These benefits include the ability to form more successful coalitions during conflict^23^ and, having friends with lots of friends themselves, allows for a more efficient transmission of information to and from others throughout a network^24^. Therefore, we could argue that there would be value for individuals to be knowledgeable about the friendships of others, and keep track of the broader friendship network of their social group as the development (or disruption) of these bonds could directly affect one’s own position in the social network and ultimately, one’s fitness.

Here, we investigated the understanding of third-party friendships in a species of macaque, the Tonkean macaque (*Macaca tonkeana*). Tonkean macaques live in highly tolerant multi-male multi-female societies^25^, characterised more so by high degrees of affiliation and communicative complexity^25,26^ and less so by strict egalitarian dominance structures. Tonkean macaques are a member of a group of closely-related Sulawesi macaque species that have been demonstrated to develop strong friendships networks with others, and in some contexts are more influenced by these friendships than those relationships built around dominance and kin^8,27^. As a consequence, the Tonkean macaque represents an ideal candidate for this study here, as we may expect any selective pressures driving the ability to understand third-party friendships to be exaggerated in this species.

To assess if the macaques could demonstrate an understanding of third-party friendship connections within their group, we designed a playback experiment. Playback experiments have been used extensively to explore third-party social knowledge in primates^4,5,13,14,28^, and much of what we know about social knowledge in primates so far, is a product of these experiments. In a similar approach to Bergman *et al*.^13^, we collected agonistic vocalisations and presented subjects with simulated conflicts between female group-mates; some of which represented a disruption of a third-party friendship, and some of which represented a disruption between non-friends. To explore if the macaques had a knowledge of not only quality of friendships between individuals, but, whether or not they could demonstrate an understanding that some third-party friendships are indirectly more beneficial to themselves than others (e.g. that it represents an important personal connection to the rest of the group^21^), we presented subjects with conflicts involving both ‘friends-of-friends’, and conflicts involving those with a greater degree of separation. Finally, to assess for the relative importance of these relationships compared with other relationship types (e.g. kin and dominance relationships), we also simulate conflicts between within and between-family individuals.

We predicted that subjects would show a stronger response (measured through looking-time towards the direction of a playback^29^) to conflicts involving close-friends compared with those involving non-friends, as these interactions represent a greater disruption to the social network and are potentially detrimental to the cohesion of the social group. We expected that this effect would be exaggerated when this conflict involved a personal friendship connection, compared to conflicts which involve no direct connections as the former may represent an important link from the subject to the rest of the social network. Finally, due to the more relaxed and tolerant social style of the Tonkean macaques, we expected that disruptions between friends would impact the responses of subjects more so than a disruption between kin or of a dominance relationship.

## Results

### Caller identity in conflict vocalisations

Here, we wanted to access whether conflict vocalisations were uniquely distinct between individuals and could be correctly classified to the caller based on their acoustic properties. Following the pDFA procedure, conflict vocalisations (gecker call units) were classified to the correct caller identity (n = 9) in 43.7% of the time, which was significantly more accurate when compared with the 5000 permuted random datasets (mean random classification accuracy: 32.1% [95% CI: 27.2–37.4%], p < 0.001). A cross-validated analysis demonstrated a similar degree of classification accuracy of 41.5% (mean random classification accuracy: 28.9% [95% CI: 23.3–35.4%], p < 0.001). On an individual basis, the linear discriminant analysis performed better than chance when classifying all nine individuals included in the analysis. A visualisation of the distribution of the permuted datasets, in addition to further details about the linear discriminants generated can be found in Supplementary Figs. SM1 and SM2. These results suggest that conflict vocalisations can be distinguished statistically based on the identity of the caller. Importantly, it provides support for the use of agonistic vocalisations in the subsequent playback experiments.

### Response to playbacks

Eighty-two playback trials were conducted on 16 individuals. Three individuals were excluded from the task, due to the fact they were never observed to be isolated from the main group (and thus, the playback criteria could not be met). Trials on the same individual were separated on average by 22.3 days (±16.4 SD). Playbacks elicited a looking response in the macaques in 74 out of 82 trials (87.8%).

**Figure 1:**
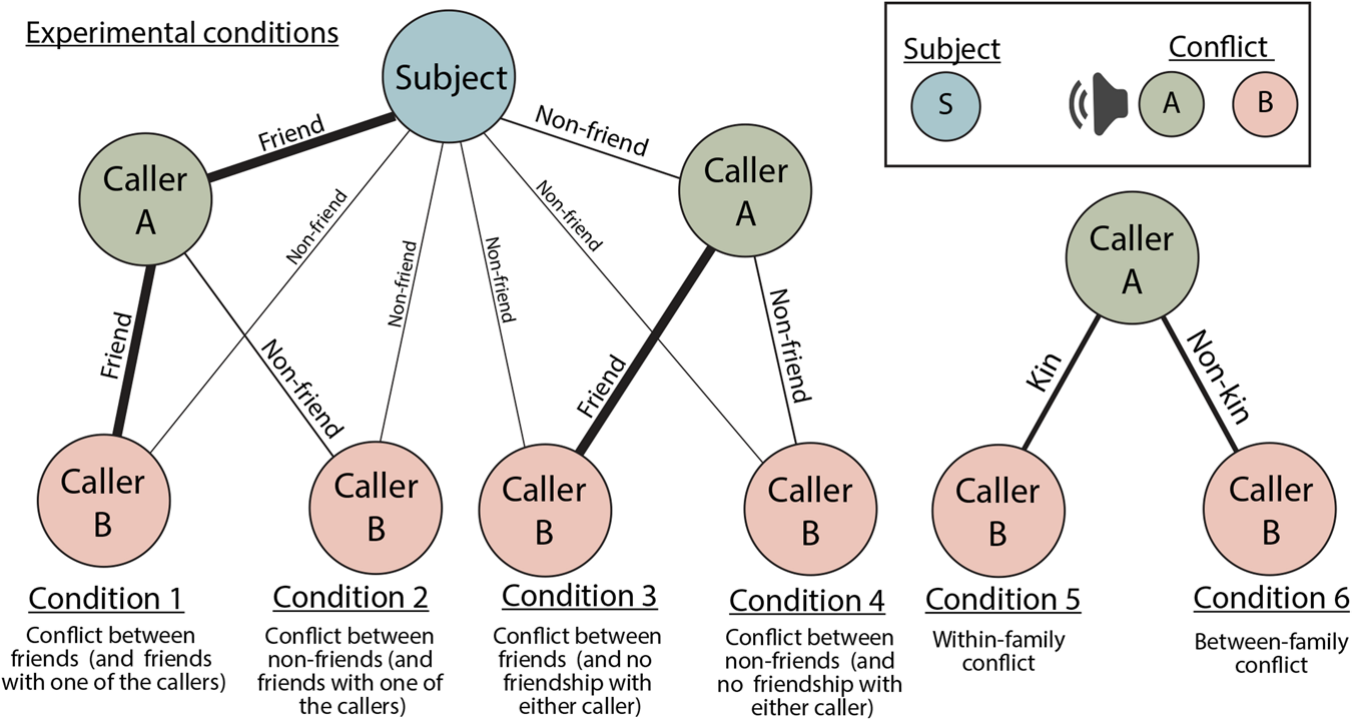
Visualisation of experimental conditions. Subjects were presented with a single playback per condition (6 playbacks in total); the composition of each stimulus is shown here. All playbacks simulated a bidirectional conflict between two females, the start-time of the two calls within a playback (Caller **A,B**) were synchronous.

Six experimental conditions were compared, all featuring a third-party conflict (Fig. 1). Two conditions included a conflict between friends (Condition 1 and 3), two included a conflict between non-friends (Condition 2 and 4), two conditions included an individual with a direct friendship with the subject (Condition 1 and 2) and two conditions involved individuals with no friendship with the subject (Condition 3 and 4). Two additional conditions featured a conflicted between kin (condition 5) and non-kin (condition 6). Friends were selected based on association data (individuals which were observed nearby (<2 m) in higher frequencies were defined as friends, and those observed nearby rarely were defined as non-friends).

**Figure 2:**
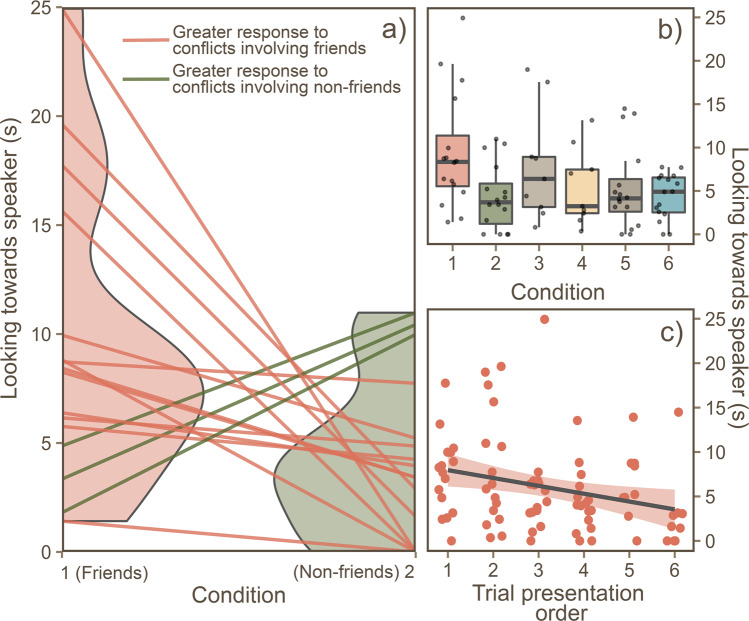
The effects of condition on the subjects’ looking time towards the speaker. (**a**) A comparison of conditions 1 and 2: subjects’ looking response was significantly higher when hearing two friends engaged in a conflict, compared with two non-friends. Half-violin plots represent the distribution of the responses, and each line represents the difference between each individual. (**b**) Boxplot comparing subjects’ looking response across all conditions. Each box represents the 25^th^, 50^th^ (median) and 75^th^ percentile. (**c**) The negative linear relationship between the total time spent looking towards the speaker and trial presentation order, each data point is an experimental trial. Figure generated using package *ggplot2* for R (^30^ open access: https://ggplot2.tidyverse.org).

Our first model (Table 1, Model 1), which looked at the effect of condition (Fig. 1) on the subjects looking time towards the speaker, was significantly improved compared with the null model (Likelihood ratio test: *X*^2^ = 8.93, df = 3, p = 0.030). Subjects in Condition 1 (conflict between friends where the subject was friends with one of the individuals, n = 16) looked significantly longer towards the speaker (9.49 s ± 1.67, mean ± SE), compared with subjects in Condition 2 (conflict between non-friends where the subject was friends with one of the individuals, Fig. 2a, n = 16, 4.30 s ± 0.94) and Condition 4 (conflict between non-friends where the subject was not friends with either individual, n = 9, 5.41 s ± 1.46). No difference in subjects’ look time was found when comparing Condition 1 to Condition 3 (a conflict between friends where the subject was not friends with either individuals, n = 9, 7.93 s ± 2.16).

**Table 1:** The effect of condition on subjects’ looking time towards the speaker.

Model	Predictor	Estimate	SE	t	*p*
1	Condition 1 (friends)	*Reference group*	—	*-—*	
	Condition 2 (non-friends)	−4.578	1.826	−2.507	***0.016***
	Condition 3 (friends)	−1.190	2.117	−0.562	0.567
	Condition 4 (non-friends)	−4.558	2.129	−2.141	***0.038***
	Trial presentation order	−1.306	0.451	−2.893	***0.006***
	Conflict intensity	0.280	0.498	0.563	0.576
2	Condition 3 (friends)	*Reference group*	—	—	
	Condition 4 (non-friends)	−3.479	2.631	−1.323	0.207
	Trial presentation order	−1.431	0.892	−1.605	0.131
	Conflict intensity	−0.013	0.863	−0.015	0.988
3	Condition 5 (within-family)	*Reference group*	—	—	
	Condition 6 (between-family)	−1.371	1.401	−0.978	0.336
	Trial presentation order	0.129	0.454	0.284	0.778
	Conflict intensity	0.739	0.532	1.388	0.176

Condition 1: A third-party conflict between friends, with a friendship connection to one of the callers. Condition 2: A third-party conflict between non-friends, with a friendship connection to one of the callers. Condition 3: A third-party conflict between friends, with a no friendship connection to either of the callers. Condition 4: A third-party conflict between non-friends, with a no friendship connection to either of the callers. Condition 5: A conflict between related individuals. Condition 6: A conflict between unrelated individuals. Significant p-values are shown in bold*.

In our second model (Table 1, Model 2), we found no difference in looking time when comparing Condition 3 and Condition 4 (Likelihood ratio test: *X*^2^ = 2.12, df = 1, p = 0.146).

In our third model (Table 1, Model 3), we also found no difference in looking time when comparing Condition 5 (conflict within-family, n = 16, 5.34 s ± 1.21) and Condition 6 (conflict between-family, n = 16, 4.39 s ± 0.65, Likelihood ratio test: *X*^2^ = 1.075, df = 1, p = 0.300, see Fig. 2b) respectively.

These findings suggest that subjects were sensitive to the friendship of the callers, looking for longer towards conflicts involving friends. Whether or not the conflict directly involved a friend of the subject may also be affecting the extent to which subjects look towards the conflict, as comparisons between Conditions 1 and 2 show a greater difference than comparisons of Conditions 3 and 4. These results also suggest that the impact of friendship on the subjects’ response to a conflict was greater than that of relatedness. Of the control factors, the intensity of the conflict in the playback had no effect on the subjects’ response. However, in our first model (Table 1, Model 1) the trial presentation order did. The response to the playbacks weakened as the amount of trials the animals were exposed to increased, suggesting that was some habituation affect due to repeat exposure to playbacks (Fig. 2c). However, we believe this not to be an issue for two reasons; first, the order of exposure to each condition was randomised between individuals so no conditions were linked to a specific trial number, second, the null model which our full model was compared with also contained trial presentation order as a fixed-effect, therefore any significant improvement of model was specifically due to the subsequent inclusion of Condition.

**Figure 3:**
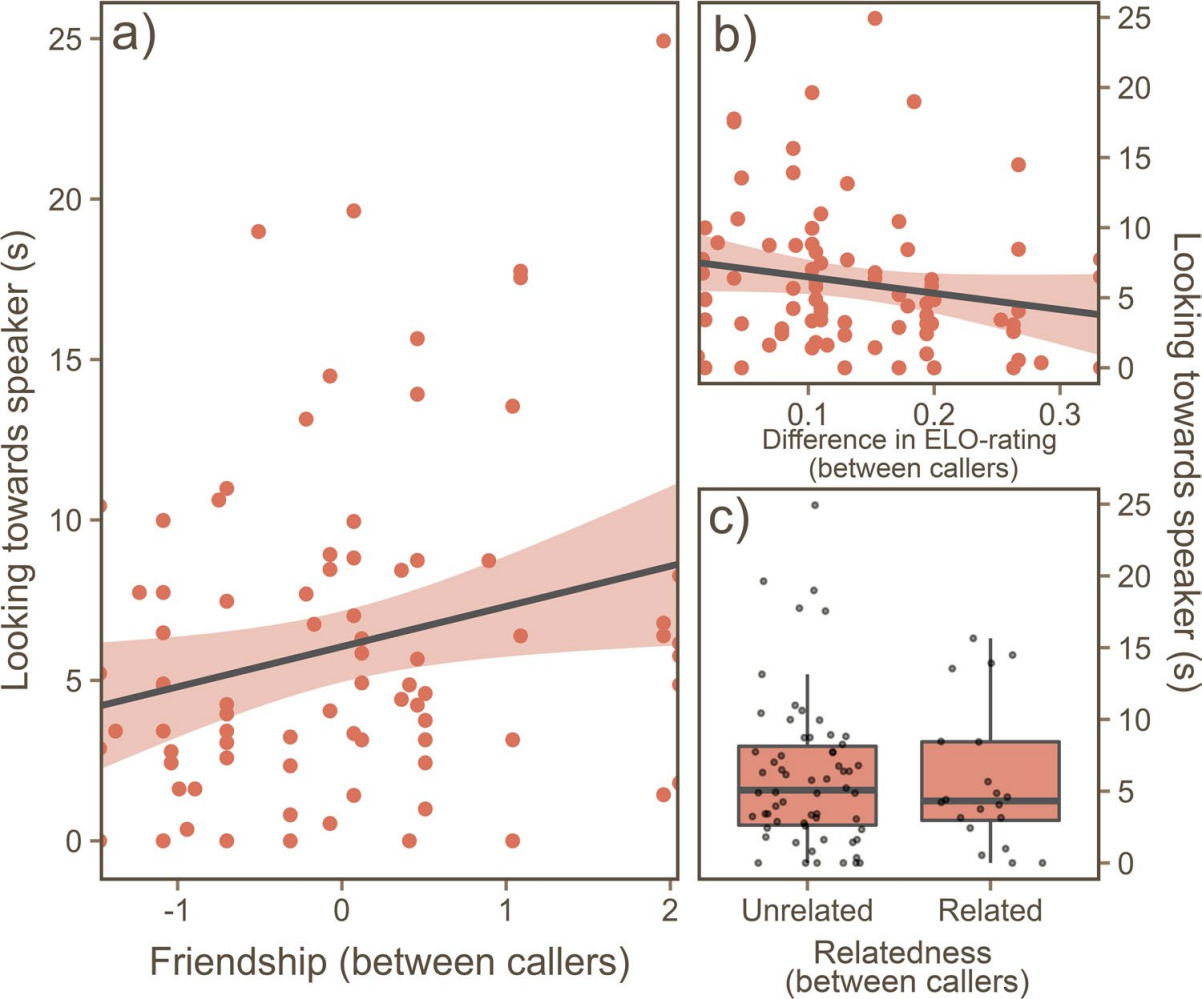
The effects of the social relationship between callers on the subjects’ look time towards the speaker. (**a**) Significant linear relationship between friendship between callers and total look time towards the speaker. Each data point is an experimental trial. On the y-axis, a higher friendship score signifies a more socially bonded dyad in the playback. (**b**) Relationship between the ELO-difference between callers and total looking time towards the speaker; a smaller ELO-difference represents individuals whom are closer together in the dominance hierarchy. (**c**) Boxplot comparing caller relatedness on the total looking time towards the speaker. Each box represents the 25^th^, 50^th^ (median) and 75^th^ percentile. Figure generated using package *ggplot2* for R (^30^ open access: https://ggplot2.tidyverse.org).

Our second model (Table 2), which looked at the effect of social relationships on the subjects looking time towards the speaker, was significantly improved compared with the null model (Likelihood ratio test: *X*^2^ = 20.24, df = 4, p = <0.001). Only a single social factor included in our model predicted the looking time in the macaques - the friendship between the two callers (Fig. 3a). The difference in competitive success (i.e. rank) and the relatedness between callers however, had no significant effect (Fig. 3b,c respectively).

**Table 2:** The effect of social relationships and attributes on subjects’ looking time towards the speaker.

Predictor	Estimate	SE	t	*p*
Friendship (between callers)	1.162	*0.572*	2.031	***0.046***
Relatedness (between callers)	−0.591	1.332	−0.447	0.656
Difference in ELO-rating (between callers)	−4.851	7.02	−0.691	0.492
Centrality of subject	−3.929	2.923	−1.344	0.183
Trial presentation order	−0.889	0.352	−2.524	***0.014***
*Response variable:*	*Looking the towards speaker (total duration)*			

Models included the random effect of subject ID. Significant p-values are shown in bold*.

## Discussion

This study provides evidence that macaques have a social knowledge regarding the quality of third-party friendships within their social group, and can use this information adaptively by selectively monitoring key social interactions. Macaques looked for longer at conflicts involving two individuals characterised by a higher friendship score (i.e. friends), compared with conflicts involving those characterised by a lower friendship score (i.e. non-friends). The relationship between the strength of a third-party friendship and looking time towards the speaker was linear, suggesting that the classification of friendships may not be as simple as a binomial ‘friend or non-friend’, but intermediately affiliated individuals were also recognised. The degree of separation between the subject and the dyad may be playing an additional role. The subjects’ looking time towards the conflicts involving friends was greater when the conflict had a direct friendship connection with the subject. This may therefore, provide evidence to suggest that the macaques were considering these relationships in terms of how they directly affected their own position in the social network, and were not just responding to a disruption to the social network as a whole. Surprisingly, the quality of friendship between the two callers in the playback was the only social variable found to modulate the response to the playbacks. A difference in rank between the callers, or, whether or not the callers were related had no impact on the macaques’ response. These results extend our understanding of social knowledge in primates, and highlight the adaptive value and the importance of friendships in primate societies.

Although a comparison of looking time is a useful quantitative measure to demonstrate that an animal is discriminating between different kinds of stimuli, it usually does not inform us about the mechanisms that underlie any looking preferences^29^. Some hypothesise that looking time is a reflection of how useful (or how adaptive) the information acquired from the stimulus is (i.e the more useful the information, the longer a subject will attend to it^31,32^), whilst others suggest that looking time is associated with a violation-of-expectancy, and primates selectively look at more unusual or novel stimulus^29^. Here, the mechanisms could be a combination of both. The macaques may be attending more so towards the conflicts occurring between friends, as these represent more unusual and novel social interactions, but also, they may be providing a useful update of social knowledge regarding the quality of the relationship (or even the structure of the social network). Whereas in contrast, the occurrence of conflicts between non-friends are perhaps more common (and less novel), and may not provide any new social information (i.e. a weak relationship would remain weak after a conflict). Or, it could be that the attention to the conflict reflects the probability that the subjects will also become involved in a conflict themselves, as the strength of social bonds between macaques reflect potential coalition partners^33^. Finally, as a word of caution, we also wish to propose an alternate explanation that the macaques’ responses are not being influenced by an understanding of friendship, but instead, an understanding of the centrality of the callers. Central individuals are more likely to have strong social bonds (‘friends’) with other central individuals, and weak bonds (‘non-friends’) with peripheral individuals. Our results therefore, could be somewhat driven by the subject responding to conflicts between two central individuals, compared with a central individual and a peripheral individual. This type of knowledge could even be a less cognitively demanding strategy, as instead of the need to remember the association between each unique dyad, an individual would only need to remember the position of each group member in the social network.

The relative dominance between each caller, and between the subject and the callers, had no influence on looking time. Similarly, we found no effect of relatedness. We predicted that a conflict between high- and low-ranking individuals (e.g. a big difference in ELO-rating) would elicit a stronger response in subjects, compared with conflicts between individuals of similar rank, as the outcome of these conflicts would represent a bigger disruption of the hierarchy. Although this somewhat contrasts other research^13,28^, we believe this could be a reflection of our stimuli composition. The conflict stimuli used in this study were bidirectional without a clear winner or loser (whereas previous research has used unidirectional conflicts^13,28^) and therefore, these interactions may be less likely to represent any clear changing of rank order. Research on tolerant species of macaques (a social style attributed to Tonkean macaques^25^) have highlighted the modulating effects that friendship has on behaviour, and have found little to no effect of dominance and rank on behaviour in comparison^8,27^. Our findings therefore may not represent an absence of social knowledge of third-party dominance and kin relationships, but instead, disruptions to these relationship types may be less important or damaging to group cohesion and thus the modulating effect of these relationships on macaques’ response to these may be more difficult to demonstrate statistically. We may expect that an experimental paradigm which assesses this social knowledge without relying on a behavioural response as evidence (e.g. an image classification paradigm such as a matching-to-sample task, or a choice task between congruent vs. incongruent stimuli^34,35^) may be more successful in demonstrating an understanding of third-party dominance and kin relationships in this species.

The findings of this research not only contribute to our understanding of third-party social knowledge, but also confirm the macaques ability to extract social information from vocalisations^36,37^. In addition, although it would be bold to directly infer the socio-cognitive mechanisms allowing for third-party social knowledge, these findings may help us to better understand the capacity for perspective-taking in monkeys^38^. If the macaques are sensitive to these differences in the social relationship quality of others, this may suggest that the animals have an understanding that others have different and unique relationships compared with their own - a potential building block for complex cognitive processing such as theory of mind^39^.

## Methods

### Subjects

We studied a social group of semi-free ranging Tonkean macaques (*Macaca tonkeana*), at the Primate Centre of the University of Strasbourg. The group consisted of 23–24 individuals during period of research: 19 adults (10 females), four juveniles and one newborn. Subjects had free access to an approximately 3700m^2^ wooded outdoor area, connected to a 20m^2^ heated indoor area. Animals were provisioned with commercial monkey pellets seven days a week, in addition to a supply of fresh fruit and vegetables once a week. Water was available *ad libitum*. Animals were highly habituated to the presence of experimenters, and although all adults had prior experience with cognitive research, all were naïve to playback experiments.

### Quantifying social relationships and attributes

To quantify social relationships within the group, behavioural data were collected between January and May 2019. Each adult (n = 19) was followed in a pseudorandom order (all individuals were followed once before moving onto a subsequent round of follows) for a 20 minutes period using focal animal sampling^40^. Every two minutes during a focal follow, a proximity scan was conducted where all nearby animals (within two meters) were recorded. Any unidirectional conflicts or displacement^41^ behaviours between any individuals were recorded *ad libitum*. Focal follows continued until 160 proximity scans per individual were recorded. All data were collected on a handheld tablet, with the software ‘Prim8’ for android (www.prim8software.com/^42^).

A degree of association (hereafter: friendship score) was calculated between all dyads, defined as the frequency in which both individuals were recorded as close neighbours (<2 m). When selecting ‘friends’ and ‘non-friends’ (Caller A) for each subject, we selected the two female group mates with the highest and lowest friendship scores with the subject (the individual with the higher friendship score with the subject becoming the friend, and the individual with the lower friendship score with the subject becoming the non-friend). When choosing the ‘friends’ of the second caller (Caller B), we chose an individual whom had a higher friendship score with Caller A, but a low friendship score with the subject. When choosing the ‘non-friends’ of the second caller (Caller B), we chose an individual with a similarly low friendship score with Caller A and the subject. In an attempt to further confirm that social proximity does in fact represent affiliative social bonds, we compared our association matrix with a grooming matrix (an affiliative social behaviour). These matrices were highly correlated (p < 0.001, Mantel test with 5000 permutations: package *ape* for R^43^), suggesting dyads whom are more often seen in close proximity, also spend more time engaged in affiliative social behaviour.

**Figure 4:**
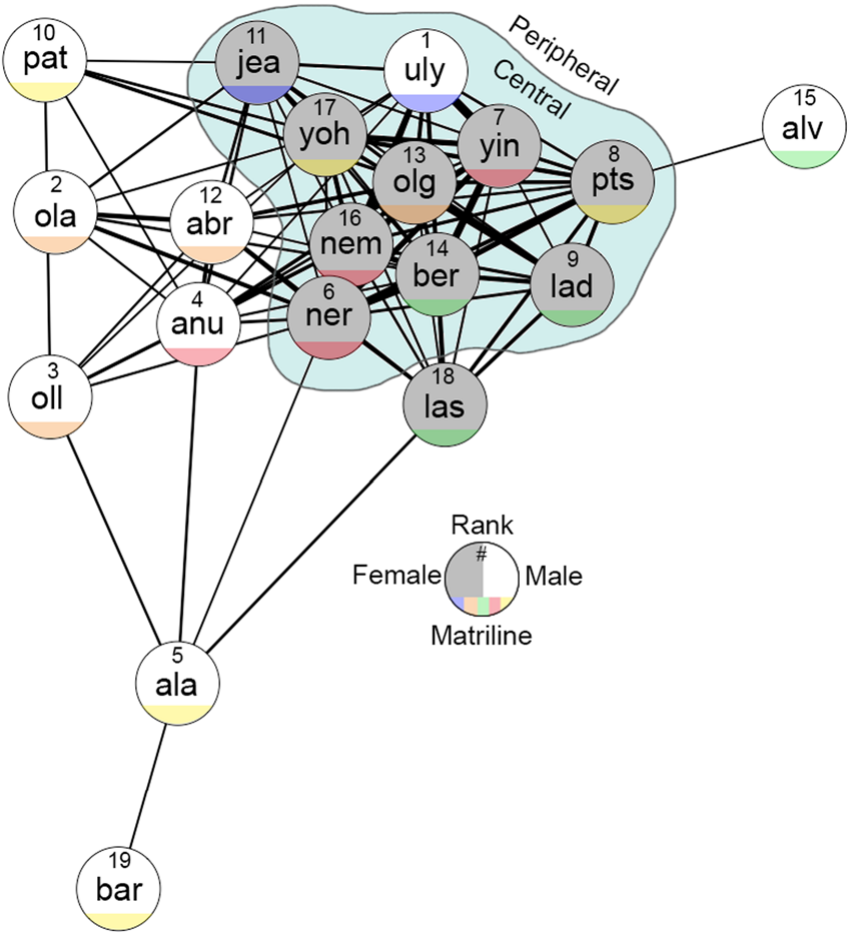
The social network of the Tonkean macaque group. A sociogram visualising the social structure of the study group where each node (circle) represents an individual in the group and each connection represents an association. The thicker the connection, and the closer the nodes are spatially, the stronger the association between the dyad. Individuals within the blue area are considered central individuals (based on eigenvector centrality), all others are considered peripheral. Absolute rank was generated by ordering ELO-ratings from high to low. To facilitate visualisation, connections were filtered, by adding edges to the sociogram until every individual had a minimum of 1 connection. Therefore, only the stronger associations are shown. Sociogram generated with package *igraph* for R (^45^, open access: https://igraph.org/r/).

All unidirectional conflicts and displacement interactions (n = 145) were used to calculate an ELO-rating for individuals (R package *EloRating*)^44^ as a measure of competitive success (or, hierarchical rank). The social group is made up of five distinct matrilines, and any individuals belonging to the same matriline were considered maternal kin (1 related, 0 unrelated). Paternal relatedness is unknown. Finally, each individual eigenvector centrality was calculated from the association data (as a proxy for social importance, using function *eigen_centrality* of the package *igraph*^45^). The half of the group with the highest centrality (n = 10) were classified as *central* individuals, the half of the group with the lowest centrality (n = 9) were classified as *peripheral* individuals. To facilitate interpretation, all friendship and dominance data were scaled to a mean of 0 and a standard deviation of 1 (Z score). A visualisation of the social group can be seen in Fig. 4.

To confirm that observed associations were in fact true relationships, we tested for stability in the social network. To do this, we subset our association data into two parts (two adjacent time blocks), and compared an association matrix generated from each subset using a matrix correlation approach (Mantel test with 5000 permutations: package *ape* for R^43^). Firstly, we compared the first 50% of the dataset with the last 50%, and second, we compared the first 95% of the dataset with the last 5%. Both pairs of association matrices were significantly correlated (p < 0.001, and p = 0.018 respectively) suggesting that social organisation of the macaques were not significantly changing over time (and associations were not random).

### Recording of conflict vocalisations and preparation of stimuli

We collected naturally occurring conflict vocalisations from the group of Tonkean macaques. All audio stimuli were recorded using a Sennheiser directional microphone (model: MKH-416-P48, frequency response: 40–20000 Hz) connected to a Tascam HD audio recorder (model: HDP2). All vocalisations were recorded at a sampling rate of 44.1 kHz and stored as a.wav file). The identity of the caller was recorded as an audio note at the end of the vocalisation. Audio clips with a low signal-to-noise ratio were discarded (e.g. excess background noise due to wind), and only recordings were a vocalisation was produced in isolation of other vocalisations (i.e. non-overlapping calls) were catalogued for further use. This resulted in approximately 117 useable calling bouts, from 14 adults.

To produce stimuli that would mimic a conflict between two group mates, the conflict vocalisations of two individuals were layered across an audio track so that the vocalisations were overlapping during playback. An example of the stimuli preparation process can be found in the electronic Supplementary Materials (ESM1). We produced conflict stimuli that featured only female-female conflicts (to reduce the chance that male sexual behaviour such as mate-guarding would bias our friendship data), and used only tonal screams and gecker calls in our production of the stimuli. This was due to the fact unidirectional conflicts featuring a clear vocal threat with a clear victim scream, were extremely rare in this group (and perhaps this species^25^) compared with bidirectional conflicts featuring screams and gecker calls. We therefore 1) did not want to produce stimuli that would be too novel to the animals, and 2) this allowed us to make use of a more extensive tonal scream/gecker audio library. Stimuli were produced in Logic Pro X, and were exported in the original recording format (.wav, sampling rate: 44.1 kHz). A low-cut filter was applied, reducing the amplitude of any frequency below 1 kHz (beyond the normal range for these call-types) to removing any excess bass due to wind. All recordings were standardised to four seconds in length and a total of 42 unique conflict stimuli were produced for the subsequent experiments. As the vocalisations stem from two unique social interactions, it was therefore difficult to control for the intensity of all interactions during the preparation of the experimental stimuli based on observational data. To mitigate this issue, a survey was distributed to primate staff and researchers where a rating of conflict intensity was acquired to be used to control for this issue during analysis (further details on this process can be found in the Supplementary Materials).

### Playbacks: Experiment design

Our study had six experimental conditions (see Fig. 1 above; results section). In the first condition (1), subjects were presented with a playback of the subjects’ friend (Caller A) engaged in a conflict with a friend (Caller B). In the second condition (2), subjects were presented with a playback of the subjects’ friend (Caller A) engaged in a conflict with a non-friend (Caller B). In the third condition (3) subjects were presented with a playback of the subjects’ non-friend (Caller A) engaged in a conflict with their friend (Caller B). In the fourth condition (4), subjects were presented with a playback of the subjects’ non-friend (Caller A) engaged in conflict with a non-friend (Caller B). In the fifth and sixth conditions, subjects were presented with playback of a matriarch engaged in a conflict with a (5) family member, or a (6) non-family member. A unique stimuli set was produced for each subject.

Data were collected on all adults. However, for conditions 3 and 4, we collected data on only peripheral individuals. This is due to the fact that for central individuals (those whom are more connected within the group), it was impossible to find examples of dyads that met the criteria for these conditions (i.e. it was difficult to find two individuals whom were non-friends of the central animals, but friends with each other). All experiments were conducted between June and October 2019 and within six months of the collection of behavioural data and audio stimuli.

### Playbacks: Experimental procedure

We attempted to conduct the playback trials in the most ecological valid conditions available in order to make the playbacks as realistic for the subjects as possible. To do this, we followed protocol where numerous conditions needed to be met before a playback could take place. First, we waited for a subject to naturally separate from the main group (>30 m), making sure they had no visual access to the callers that feature in the next playback stimulus. We also ensured that both callers in the playback were in roughly the same direction relative to the subject. Once these conditions were met, a speaker (JBL outdoor speaker, frequency response: 70–20,000 Hz) was set up and secured in a concealed location, 10–15 m away from the subject and in the direction of the individuals whose call is to be played. We also made sure at this point that the speaker was >25 m away from the individuals in the playback. The subject was then video-recorded (Canon Legria HFG30). Once at least one minute of inactivity was observed in the subject, and, when the subject was facing away from the speaker, the experimenter initiated the playback. Here, the audio was wirelessly transmitted to the speaker via an android device. Wherever possible, trials were video-recorded at an angle roughly perpendicular (50–130**°**) to the subject and the speaker to minimise the amount social stimulus (i.e. the experimenter) in direction of the playback. Finally, the subject was video-recorded for 30 seconds after the playback for a subsequent analysis of their response.

Experimental trials were conducted opportunistically as subjects split from the group. However, the order in which each individual was exposed to the conditions was randomised. If the next scheduled trial was not possible (e.g. the animals in the playback were not in the same direction from the subject), this trial was skipped and the next possible trial was conducted, returning to the skipped trial at the next available opportunity. To reduce a habituation response to the playback audio, experimental trials conducted on the same subject were separated by at least three days (on a single occasion we conduced a trial two days after a previous trial to take advantage of a rare occurrence, however the separation between trials were normally much longer), all trials were separated by at least 90 minutes on any given day, and a maximum of 3 trials per day were conducted. It is likely that in some cases, non-experimental individuals were able to eavesdrop on the experimental trials of others from a distance (scream vocalisations can be heard from >50 m away), and therefore it is difficult to know how much exposure any one individual has to the playback of experimental stimuli. We attempt to address this in our analyses by assessing how the strength of responses changes over the course of the experiment and we remain cautious.

### Video Coding

The responses to the experimental stimuli were measured using BORIS (Behavioral Observation Research Interactive Software^46^). In the 30 seconds following the onset of the playback, we recorded the total time the subjected spent looking towards the speaker. Approaches towards the speaker as a response were not frequent enough for subsequent analysis (observed in 16 of the 82 experimental trials), and therefore we focused only on looking behaviours. All videos were coded blind by JW (the experimental condition the video belonged to was not known at the time of video-coding). For the purpose of testing inter-rater reliability; a naïve researcher coded the subjects’ looking time in 20% of the videos, which was in significant agreement with the main dataset (Pearson’s correlation: r = 0.879, t = 6.660, p = <0.001). Examples of the videos collected during the experimental trials can be found in the Supplementary Materials (ESM2).

### Statistical analysis: acoustic analysis

**Figure 5:**
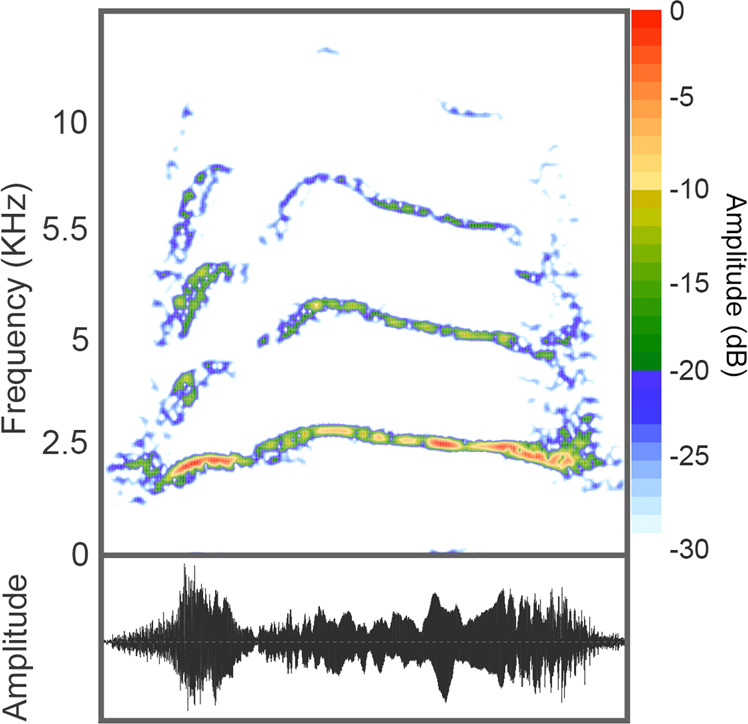
A spectrogram and waveform of an agonistic vocalisation observed in the Tonkean Macaques. Pictured is an example of a single Gecker (or squeak) call unit, a tonal agonistic call usually occurring in a repetitive sequence of multiple units. Most of the energy in this call can be found between 2 and 10KHz. Spectrograms were produced using package *Seewave* for R (FFT length = 512 points; window = hamming; overlap = 95%^50^, open access: https://cran.r-project.org/web/packages/seewave/index.html).

To assess that conflict vocalisations are individually unique in this species, we conducted a permuted discriminant function analysis procedure (pDFA,^47^). This analysis was applied to 414 gecker call units (Fig. 5,^48^), from 52 calling bouts, given by nine individuals. Each calling bout contained between two and 47 individual units, with an average of 7.96 units per bout. Gecker calls were chosen for this analysis (in opposed to other agonistic call types, e.g. tonal screams or barks^48^) as they were the most common call type that we observed and recorded (in terms of both calling bouts, and individual units per bout) and therefore allowed for the most robust acoustic analysis. Nine adult individuals (out of a potential 19) met the inclusion criteria of the analysis (the number of paramaters included in the discriminant function analysis must be smaller than the number of units in the smallest class of objects; more information regarding the pDFA procedure below). All call units were extracted in their original recording format (.wav, sampling rate: 44.1 kHz) using Praat acoustic software^49^.

For each call unit, five acoustic parameters were extracted; DFB1: first dominant frequency band; DFA2: the mean of the frequencies at which in each time segment the mean value of energy distribution was reached; PF: overall peak frequency; FR: frequency range; duration: the length of the call in milliseconds. These parameters are common for acoustic analysis in primates, and have been used successfully in the acoustic analysis of closely related species of macaques^8,51^. All parameters were extracted automatically using the *seewave* packages for R^50^ and when necessary, were log-transformed to a normal distribution. Finally, all parameters were scaled to a mean of 0, and a standard deviation of 1 before analysis. More information regarding the extracted parameters can be found in the Supplementary Materials (Table SM1).

A linear discriminant analysis (function *lda* from the R package *MASS*^52^) was applied, to assess the accuracy in which each call unit could be classified to their caller (n = 9) based on the five extracted acoustic parameters. To test whether the classification accuracy was significantly above a level of chance we applied a pDFA procedure. In this procedure, permutations of the original dataset (n = 5000) were generated by assigning each calling unit to a random caller ID, however, keeping non-independent data points nested together (in this case, our data was nested into calling bouts, and therefore call units belonging to the same calling bout were non-independent). This permutation procedure allows the analysis to accounts for nested data which could otherwise lead to an exaggerated interpretation of classification accuracy^47^. A linear discriminant analysis was subsequently applied to each of the random permutations, and the classification accuracy of the random permutations was then compared to that of the original data. If the classification accuracy of the original dataset is higher than 95% of the permutated datasets, we can conclude that the classification accuracy is significantly above chance (at p < 0.05). For more information on this procedure, please see Mundry and Sommer^47^. Finally, as a validation of the original classification, this analysis was repeated with a K-fold cross-validation procedure, where the linear discriminant analysis is trained on all data but a single point, and a prediction is made for that unseen point. All permutations were automatically generated in R.

### Statistical analysis: playback experiments

We used a generalised linear model approach to assess which factors best predicted the subjects’ response to the playbacks. All models included the total duration subjects were *looking towards the speaker* (the 30 seconds post-playback observation) as the response variable. The first models focused on the influence of experimental condition, and included the predictors; ‘condition’ in addition to two control variables, ‘conflict intensity’ and ‘trial order’. Three models where produced; one which compared condition 1 with conditions 2, 3 and 4, one which compared condition 3 with condition 4, and one which compared condition 5 with 6. An additional model focused on social relationships and attributes and included ‘the friendship between the two callers’, ‘the relatedness of the two callers, ‘the difference in competitive success between the callers’, and ‘the eigenvector centrality of the subject’. This second model also included control variables, ‘trial order’ (conflict intensity was not included here as we observed no effect of this variable in the first model), and subject ID as a random effect.

Models were built using *glm* and *lmer* functions (package *lme4)* for R. Each model was first compared with a null model, containing only the control variables and random effects (likelihood ratio test, function: *lrtest*, package: *lmtest*). If the full model was a significant improvement on the null model, the influence of each individual predictor was explored in more detail. Before any interpretation of model results, we made sure predictors did not violate any multicollinearity assumptions (by calculating a variance inflation factor for each predictor using function *VIF* in package *car*). Additionally, model residuals were visually inspected for any extreme deviations from normality (by inspecting Normal QQ-plots using function *plot*). All VIF scores were low (<1.5) for predictors in all models, and no extreme deviations from normality were observed.

### Ethical note

This research was approved by the Ethics Committee of the Ministère de l’Enseignement supérieur de la Recherche et de l’Innovation (Approval number: B6732636. Authorisation reference:APAFIS#3371-2015123015405349 v3). In addition, all methods are in compliance with the EU Directive 2010/63/EU and the ASAB/ABS guidelines for the use of animals in research.

## Supplementary information

Supplementary information

Supplementary information 1

Supplementary information 2

## Data Availability

Data and code is available on the Open Science Framework and be accessed here: 10.17605/OSF.IO/KXJ5R.
