# Supplementary material for: An understanding of third-party friendships in a tolerant macaque
Source: Sci Rep. 2020 Jun 17;10:9777. doi: 10.1038/s41598-020-66407-w (PMC7300006; doi:10.1038/s41598-020-66407-w)

Supplementary materials

An understanding of third-party friendships in a tolerant macaque

Jamie Whitehouse & Hélène Meunier

Measuring intensity of conflict stimuli

Controlling for the intensity of the conflict interaction in this study is important, as such intensity may significantly affect the likelihood of a subject to attend to the interaction. For example, high-intensity conflicts may represent a bigger social disruption regardless of whether the conflict contained a friend or otherwise. As the conflict stimuli are created from vocalisations recorded from two unique social interactions, it is difficult to quantify the intensity of the artificial conflict based on behavioural observations alone. To mitigate this, and to be able to control for conflict intensity in subsequent analyses, a voluntary survey was distributed to primate staff and peers where an accurate rating of conflict intensity was acquired. The survey consisted of questions related to the participants' professional experience with primates (e.g. '*Do you work with primates professionally and/or in research?*'; '*if yes, how long have you worked with primates?*'; '*How familiar are you with macaque vocalisations?*'), followed by a task whereby they had to listen to, and rate, all audio conflict stimuli used in the experiment. Surveys were built and distributed through the online survey interface *Jotform* (http://www.jotform.com/).

All conflict stimuli (n=42) were rated on a scale on 0 (mild) to 10 (intense). Additionally, participants had to rate 8 of the audio clips a second time as a control, to test of intra-rater reliability. This created a total of 50 audio clips to be rated for each participant. Participants were not informed that they would be required to rate some audio clips twice, and did not know at the time which clips were repeated. If the participant declared they had no prior experience in primates, or, if the correlation co-efficient between their original ratings and second control ratings was below 0.7, their ratings were discarded.

Twenty-five participants completed the survey, 20 of which met the criteria for inclusion. The average correlation coefficient between the original ratings and second control ratings for the remaining participants was 0.89. The ratings of these participants were averaged, and used as our rating of intensity for each of the stimuli.

TABLE SM1
Descriptive statistics of the extracted parameters from the Gecker Call units

ID	N	Duration	DFB	FR	PF	DFA2
Anubis	36	0.055±0.011	2.043±0.869	7.964±2.028	3.201±0.618	3.251±0.569
Berenice	44	0.074±0.031	2.660±2.660	9.953±2.651	4.525±0.849	4.642±0.896
Lady	84	0.044±0.019	2.559±1.170	8.666±1.633	3.868±0.509	3.865±0.485
Lassa	111	0.050±0.013	2.522±1.105	7.877±1.661	3.816±0.625	3.864±0.659
Nema	15	0.082±0.032	2.195±1.193	8.746±2.907	3.867±0.681	3.802±0.663
Nereis	18	0.078±0.023	2.538±0.304	8.213±0.847	3.299±0.540	3.325±0.655
Patsy	33	0.031±0.009	2.649±1.381	10.561±2.406	4.328±0.779	4.434±0.888
Yin	17	0.080±0.148	2.455±0.908	8.720±1.286	3.581±0.506	3.662±0.643
Yoh	56	0.050±0.017	2.807±1.389	9.825±2.325	3.979±0.834	4.027±0.723

All values are Mean±SD.

Figure SM1

A visualisation of the 5000 randomised datasets for the permuted discriminant analysis procedure. a) Original classifications and b) cross-validated classifications. Each histogram is marked with the required classification accuracy to achieve significance (dotted vertical line), as well as the actual classification accuracy of the dataset (solid vertical line). Figure generated using package *ggplot2* for R (https://ggplot2.tidyverse.org).

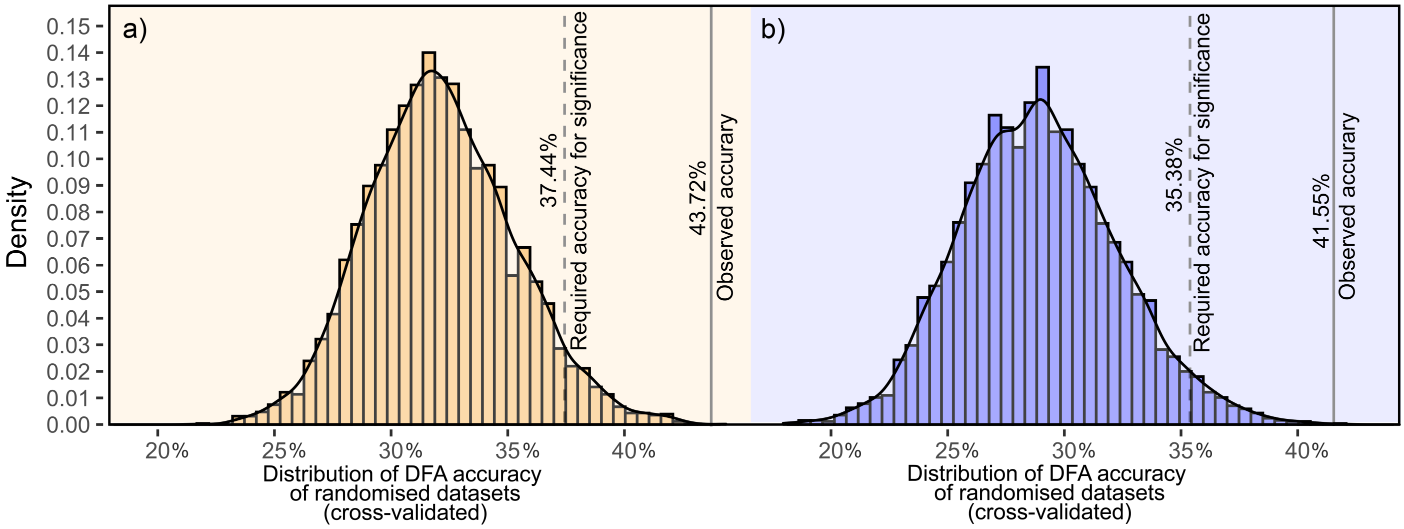

Figure SM2

Visualisation of the linear discriminants (LD) produced from the analysis of the acoustic data. LDA1, LDA2, and LDA3 account for 53%, 34% and 9% of the variance respectively. LDA4 and LDA5 (not shown) account for 2.5% of the variance. Each colour represents each classification (Caller ID). Each axis presents the loading of each LD. Scatter plots show comparisons between LD's, and histograms show the distribution of each LD singularly. Figure generated using package *ggplot2* for R (https://ggplot2.tidyverse.org).

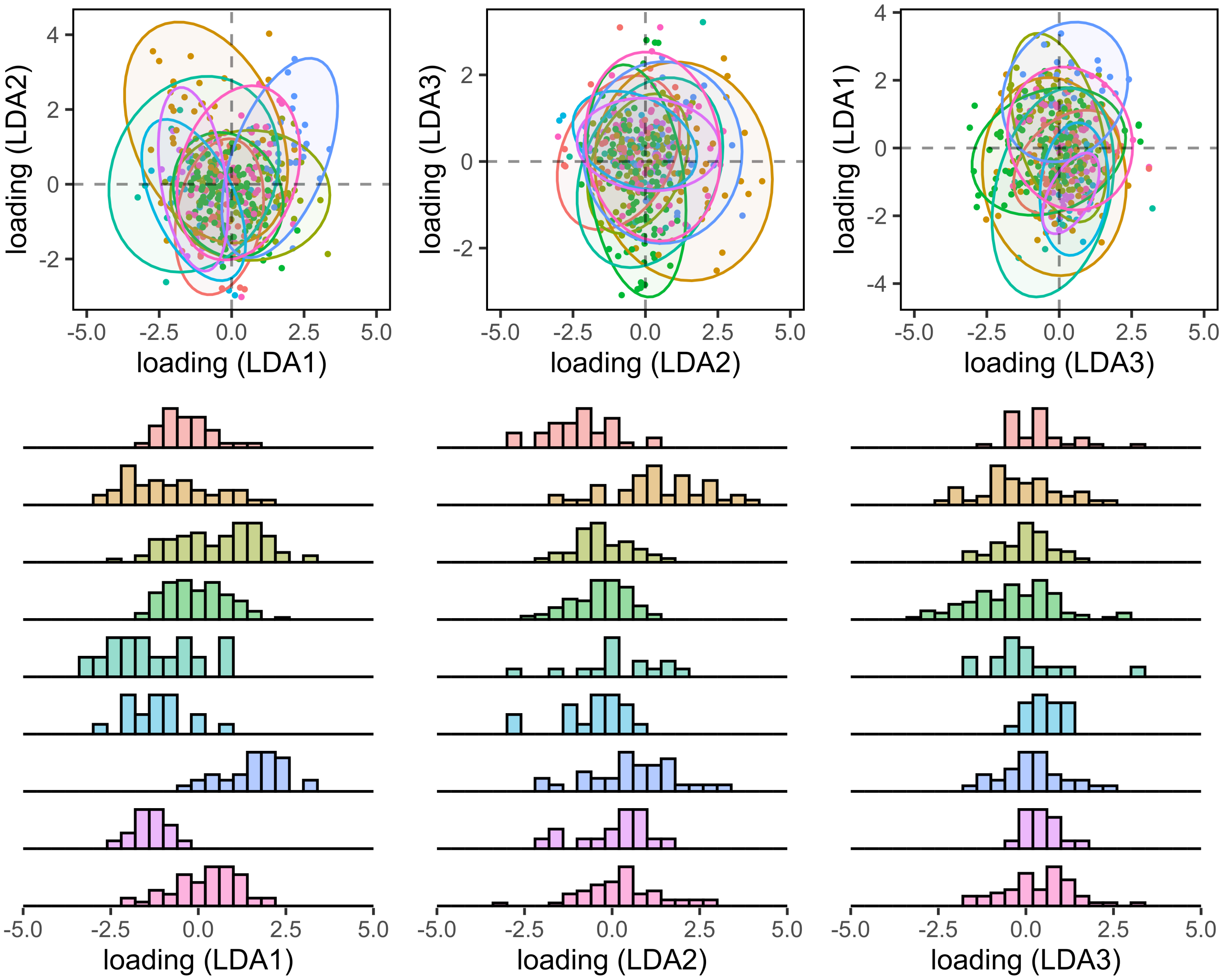

Supplement: Supplementary file 3 — Supplementary information 2 [file 41598_2020_66407_MOESM3_ESM.pdf]
